# Persistent physical and mental health effects of COVID-19 hospitalization

**DOI:** 10.3389/fped.2026.1710900

**Published:** 2026-07-30

**Authors:** David L. Goldman, Valerie Fong-Silva, Salih Demirhan, Jassour Alrikaby, Kiriam Escobar Lee, Catherine Sancimino, Sophie Molholm, Michael D. Cabana, Betsy C. Herold

**Affiliations:** 1 Division of Infectious Diseases, Department of Pediatrics, Albert Einstein College of Medicine, Children’s Hospital at Montefiore, Bronx, NY, United States; 2 Cognitive Neurophysiology Laboratory, Department of Pediatrics, Albert Einstein College of Medicine, Bronx, NY, United States; 3 Dominic P. Purpura Department of Neuroscience, Albert Einstein College of Medicine, Bronx, NY, United States; 4 Department of Pediatrics, Albert Einstein College of Medicine, Children’s Hospital at Montefiore, Bronx, NY, United States

**Keywords:** children, hospitalization, long COVID, mental health, pandemic

## Abstract

**Introduction:**

The long-term health effects of the COVID pandemic on children remain an area of active investigation. Viral infection, immune responses, and societal disruption have been implicated as contributing factors.

**Methods:**

We conducted a retrospective survey-based study of children infected with SARS-CoV-2 and uninfected controls admitted to a single urban children's hospital in the Bronx, NY, USA, during the Original/Alpha variant wave (1 March 2020 to 1 March 2021) or Omicron wave (2 December 2021 to 21 December 2022). Persistent symptoms, time to recovery, family stress, and psychiatric outcomes were assessed. A psychometric assessment using Patient Health Questionnaire (PHQ-9) and Generalized Anxiety Disorder (GAD-7) (*n* = 48) and Pediatric Symptom Checklist (PSC-17) (*n* = 64) was completed.

**Results:**

A total of 176 children and families completed surveys. Approximately 31.9% of children admitted during the Original/Alpha wave and 8.8% during the Omicron wave reported that their illness took several months or more to resolve. The most common symptoms after hospitalization were fatigue, shortness of breath, and difficulty sleeping, and these were similar to those experienced by uninfected controls. PHQ-9 and GAD-7 scores did not differ by infection status. However, PHQ-9 scores were significantly higher during the Original/Alpha wave than during the Omicron wave (median 5 vs. 1; *p* = 0.04; *r* = 0.62), with GAD-7 trending similarly (*p* = 0.05). Overall, 43.8% of children screened positive for depressive symptoms on PHQ-9, including 20.9% in the moderate-to-severe range. PSC-17 scores did not differ significantly by wave or infection status. High levels of pandemic-related stress, including job loss, childcare disruption, and food insecurity, were reported across both COVID-positive and uninfected families.

**Conclusion:**

Children hospitalized during the Original/Alpha wave experienced a significantly greater burden of prolonged recovery than those during the Omicron wave, implicating virologic and immunologic factors in postacute physical outcomes. Notably, posthospitalization symptoms were similar between COVID-positive children and uninfected controls, underscoring the importance of contemporaneous control groups in studies of postacute sequelae. Mental health outcomes were more strongly associated with the pandemic phase than infection status, suggesting societal disruption rather than direct viral effects as a key contributor. These findings highlight the need for routine mental health screening in hospitalized children during public health emergencies.

## Introduction

The COVID-19 pandemic has had a profound impact on children, but the precise long-term physical and mental health effects have not been fully understood. A recent systematic review of pediatric studies reported that approximately 16.2% of children experienced one or more symptoms for more than three months after COVID, also known as long COVID (1). There is, however, considerable variation in the reported incidence of persistent symptoms, ranging from 0.7% to70% (2, 3). This variability likely relates to several factors such as important differences in methodology, study populations, and whether controls were included. Social and economic factors are critical modifiers of the incidence and severity of both acute and long COVID. Indigent and minority populations have been reported to experience worse outcomes, including higher rates of mortality and persistent symptoms, although most of these data are limited to adults (4–7). Further complicating the understanding of COVID outcomes in children has been changes in the definition of long COVID [reviewed in (8)].

In addition to its long-term physical effects, substantial negative effects on the mental health of children have been reported in association with the COVID pandemic, with some studies reporting a doubling in the incidence of depression and anxiety compared with prepandemic years (9–11). The basis for this increase in mental disturbance has been hypothesized to stem from a variety of factors, including disruptions in routine, social isolation, increased screen time, and family stress (12–15). Low socioeconomic conditions have also been associated with worse mental health outcomes in both adults and children (16, 17). In this regard, COVID-related hardships (i.e., income loss and increased caregiving requirements) have been linked to poor family psychological wellbeing (15), although some studies have found that mental health outcomes such as depression and anxiety may be worse among children from higher-income families (18, 19).

To address some of the gaps in understanding the long-term impact of COVID in children, we surveyed families who had a child hospitalized at a single urban children's hospital that serves a demographically diverse population in the Bronx, NY, USA. We compared children hospitalized with COVID with those hospitalized during the same period with non-COVID-related illnesses, focusing on persistent physical and mental health outcomes and perceived stress. To provide additional perspective, we also compared the health outcomes for patients hospitalized with COVID during the early and later phases of the pandemic.

## Materials and methods

### Study design and definitions

We conducted a retrospective cohort study of patients admitted to the Children's Hospital at Montefiore during either the Original/Alpha variant wave of SARS-CoV-2 infection (1 March 2020 to 1 March 2021) or the Omicron wave (2 December 2021 to 21 December 2022). The inclusion criteria for cases were (1) admission to the Children's hospital during the specified wave periods and (2) a positive SARS-CoV-2 molecular assay at the time of admission, regardless of whether COVID-19 was the primary reason for hospitalization. The inclusion criteria for controls were (1) admission during the same periods and (2) a negative SARS-CoV-2 molecular assay at admission. The exclusion criteria for all participants were (1) inability of the guardian to complete a survey in English or Spanish and (2) death prior to the survey period. Controls were matched to cases on admission date (within 3 months), age, and presence of a chronic medical condition (e.g., cancer or sickle cell disease). Surveys were administered 9–12 months following the index admission.

Patients or their parent(s) were contacted by phone for recruitment into the study. Following obtaining oral consent and assent when appropriate, patients were enrolled in the study and provided with a link to complete an online survey. Attempts to contact patients for potential enrollment were made on three separate occasions, 1 week apart, either by phone or by mobile telephone short message service (i.e., text messaging). For their participation, their families were sent a gift card worth $25. This study was approved by the Institutional Review Board at the Albert Einstein College of Medicine.

### Parent and patient surveys

Surveys were created on the RedCap platform and contained questions related to demographics and subsequent healthcare utilization including emergency department (ED) and urgent care visits and hospitalizations (see Supplementary Appendix) (20, 21). Two complementary but distinct assessments of postdischarge illness burden were included. Parents of children with COVID reported overall time to full recovery using ordinal response categories ranging from days to more than one year. Parents and patients in both groups additionally identified postdischarge symptoms and characterized their duration using the same categories. Long COVID was operationally defined as reported recovery time of several months or greater, consistent with CDC guidance defining long COVID as persisting at least 3 months after acute infection (22).

### Psychometric testing

Children over 11 years of age were asked to complete a Generalized Anxiety Disorder scale (GAD-7) and a modified Patient Health Questionnaire (PHQ-9) (23–25). The PHQ-9 score was modified by removing the question related to suicide. Removal of this item reduces the maximum possible score from 27 to 24. This modification was made to ensure survey appropriateness for this pediatric population and has been used in prior pediatric studies in clinical settings (26). Parents were asked to complete a Pediatric Symptom Checklist (PSC-17) for children 4–11 years of age (27, 28). Parents and patients were also queried about a variety of psychosocial stress, assessed via Likert-scale items addressing pandemic-related stressors that included food insecurity, loss of income, housing instability, childcare disruption, and social isolation (29).

### Data analysis

Statistical analyses were conducted using Stata version 17.0 (StataCorp LLC, College Station, TX, USA) and Graph Pad Prism 10 (Boston, MA, USA). Comparisons were made between cases and controls as well as between children infected during the early (presumed Original/Alpha variant) and later (presumed Omicron) waves. The distribution of continuous variables was assessed both visually with histograms and statistically with skewness and kurtosis tests. Non-normally distributed continuous variables were presented as median [interquartile range (IQR): 25%–75%], whereas categorical variables were presented as counts (percentages). Continuous variables were tested for normality and Mann–Whitney *U* or Student's *t*-tests were used accordingly. Fisher’s exact test or a Chi-squared test was used for comparing categorical variables.

Power calculations were conducted using asymptotic relative efficiency and effect size was calculated using Rank-Biserial Correlation®. To evaluate for potential confounding of psychometric outcomes, bivariate analyses were performed examining associations between PHQ-9, GAD-7, and PSC-17 scores and key clinical and demographic variables, including sex, age, intensive care unit (ICU) admission, multisystem inflammatory syndrome in children (MIS-C) status, and length of stay. Given the absence of statistically significant bivariate associations and the limited sample size, a multivariable regression analysis was not performed. Likert-scale stress responses were collected using a five-point scale ranging from Strongly Disagree to Strongly Agree and collapsed into three categories for analysis: disagree, neutral, and agree. This approach was chosen to ensure adequate cell counts for Chi-squared testing given our sample size and to facilitate comparison across domains. *p*-Values <0.05 were considered statistically significant.

## Results

### Demographics

For the Original/Alpha wave, 162 infected and 117 non-infected controls were contacted, with 56 (34.6%) infected and 30 (25.6%) uninfected completing their surveys entirely. For the Omicron wave, 124 infected and 118 uninfected were contacted, with 46 (37.1%) infected and 44 (37.3%) uninfected (36.4%) completing their surveys entirely. The overall response rate was 34% (176/521). There were an additional eight survey responses (three COVID and five controls) that provided partial responses to some long-term health follow-up questions but provided complete responses to PSC-surveys. These data were included within the PSC-analysis only. There were 10 individuals who were older than 21 years (range 22–24) in the Original/Alpha wave. There were no differences in the median age, male:female ratio, ethnicity, presence of chronic conditions, or race between cases and controls (Table 1). The overall length of stay for children with COVID was longer than that for controls (3 vs. 2 days, *p* < 0.001). Compared with children infected with the Omicron variant, children infected with the Original/Alpha variant were older, more likely to have MIS-C, more likely to be admitted to the ICU, and to have longer lengths of stay (Table 2). The distribution of pre-existing psychiatric/behavioral and medical comorbidities did not differ significantly between the Original/Alpha and Omicron groups, although medical comorbidities trended higher in the Omicron cohort (58.7% vs. 39.3%, *p* = 0.07). In addition, all children with the Original/Alpha strain were admitted for COVID-related illness, whereas only 19/46 (41.3%) of those infected with Omicron were admitted for COVID-related illnesses.

**Table 1:** Demographics and clinical features of children with COVID and controls.

Features	All *n* = 176	Cases *n* = 102	Controls *n* = 74	*p*-Value
Age, years (median, IQR)	6 (2–16)	8 (2–16)	4.5 (2–14)	0.2
Sex, male	98 (55.7)	55 (53.9)	43 (58.1)	0.6
Race				0.1
White	26 (14.8)	15 (14.7)	11 (14.9)	
Black	60 (34.1)	37 (36.3)	23 (31.1)	
Asian	6 (3.4)	1 (1.0)	5 (6.8)	
Other	73 (41.5)	41 (40.2)	32 (43.2)	
Not reported	11 (6.3)	8 (7.8)	3 (4.1)	
Ethnicity				0.7
Hispanic	83 (47.2)	46 (45.1)	37 (50.0)	
Non-Hispanic	73 (41.5)	45 (44.1)	28 (37.8)	
Not reported	20 (11.4)	11 (10.8)	9 (12.2)	
Strain				
Alpha	56 (31.8)	56 (54.9)		
Omicron	46 (26.1)	46 (45.1)		
Pre-existing condition	92 (52.3)	57 (55.9)	35 (47.3)	0.3
Psychiatric/behavioral	8 (4.5)	6 (5.9)	2 (2.7)	0.5
Medical	82 (46.6)	50 (49.0)	32 (43.2)	0.4
MISC	15 (8.5)	15 (14.7)	0	0.001
ICU admission	46 (26.3)	29 (28.4)	17 (23.3)	0.4
Length of stay, days (median, IQR)	2 (1–4.5)	3 (2–5)	2 (1–3)	<0.001

Unless indicated, numbers of patients are shown with percentages in parentheses. *P*-values represent a comparison between patients with COVID and controls. Pre-existing condition categories: Psychiatric/Behavioral (*n* = 8) includes behavioral disorders (*n* = 5) and psychiatric diagnoses (*n* = 3). Pre-existing medical conditions include sickle cell disease (*n* = 25), asthma (*n* = 18), congenital heart disease (*n* = 7), diabetes mellitus (*n* = 6), neurologic/neuromuscular/developmental delay (*n* = 5), chronic renal disease (*n* = 4), obesity (*n* = 3), and syndromic conditions (*n* = 3); the remaining conditions each have *n* ≤ 2. .

**Table 2:** Demographics and clinical features of COVID-hospitalized patients stratified by variant era (Original/Alpha vs. Omicron).

Features	All (*n* = 102)	Alpha (*n* = 56)	Omicron (*n* = 46)	*p*-Value
Age, years (median, IQR)	8 (2–16)	8.5 (3.5–20)	4 (1–14)	0.005
Sex, male	55 (53.9)	30 (53.6)	25 (54.4)	0.9
Race				0.07
White	15 (14.7)	5 (8.9)	10 (21.7)	
Black	37 (36.3)	17 (30.4)	20 (43.5)	
Asian	1 (1.0)	0	1 (2.2)	
Other	41 (40.2)	28 (50.0)	13 (28.3)	
Not reported	8 (7.8)	6 (10.7)	2 (4.4)	
Ethnicity				0.3
Hispanic	46 (45.1)	26 (46.4)	20 (43.5)	
Non-Hispanic	45 (44.1)	22 (39.3)	23 (50.0)	
Not reported	11 (10.8)	8 (14.3)	3 (6.5)	
Pre-existing condition	57 (55.9)	27 (48.2)	28 (63.6)	0.23
Psychiatric/behavioral	6 (5.9)	5 (8.9)	1 (2.2)	0.22
Medical	49 (48.0)	22 (39.3)	27 (58.7)	0.07
MISC	15 (14.7)	13 (23.2)	2 (4.4)	0.007
ICU admission	29 (28.4)	21 (37.5)	8 (17.4)	0.03
Length of stay, days (median, IQR)	3 (2–5)	4.5 (2–8)	2 (1–3)	<0.001
COVID-related admission^a^	75 (73.5)	56 (100.0)	19 (41.3)	<0.001

Unless otherwise indicated, numbers of patients are shown with percentages in parentheses. Continuous variables were compared using the Mann–Whitney *U* test; categorical variables were compared using the chi-squared or Fisher's exact test as appropriate. *p*-Values represent comparisons between Original/Alpha and Omicron groups.

a All patients in this cohort tested positive for SARS-CoV-2; this variable indicates whether COVID-19 was determined to be the primary reason for hospitalization, as opposed to incidental detection.

### Symptoms and recovery

**Figure 1:**
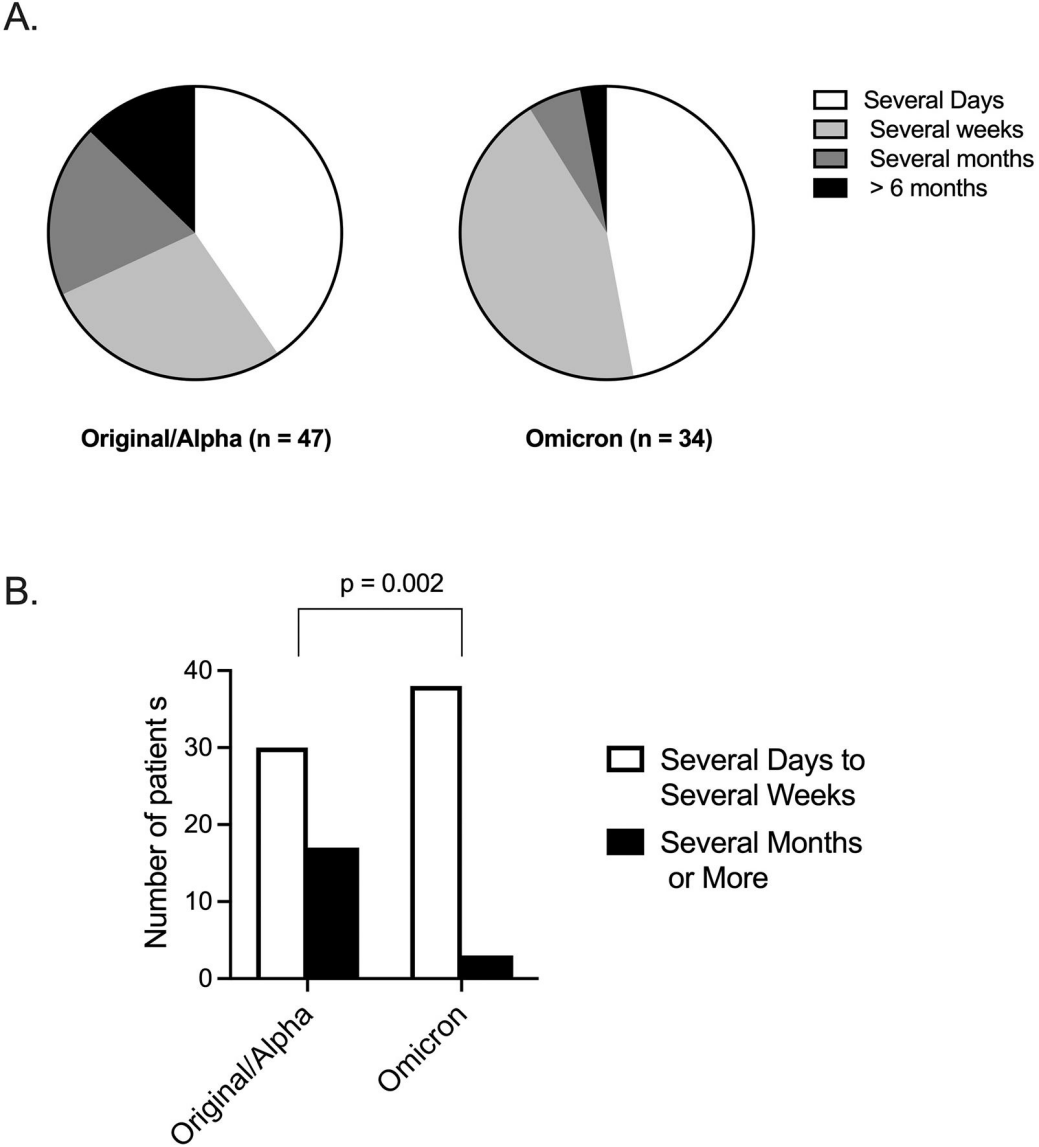
Time to recovery following COVID-19 hospitalization by pandemic wave. **(A)** Distribution of time to recovery among COVID-19-positive children admitted during the Original/Alpha and Omicron variant waves, expressed as a percentage of each group. **(B)** A bar chart showing a comparison of the number of children reporting short-term recovery (several days to several weeks) vs. prolonged recovery (several months or more) between the Original/Alpha and Omicron waves. Children admitted during the Original/Alpha wave were significantly more likely to report prolonged recovery compared with those admitted during the Omicron wave (*p* = 0.02).

The majority (63/81, 77.8%) of children with COVID reported recovery from their illness within days to several weeks following infection, while the remaining 18/81 (22.2%) reported a more protracted illness, with time to full recovery of several months or more, meeting the study's operational definition of long COVID. Among these 18 patients, 10 (55.5%) were male with a median age of 13 years (range 3–24 years). A greater proportion of patients infected with the Original/Alpha variant (31.9%, 15/47) reported a recovery time of several months or more compared with those infected with Omicron (8.8%, 3/34, *p* = 0.02) (Figure 1).

**Figure 2:**
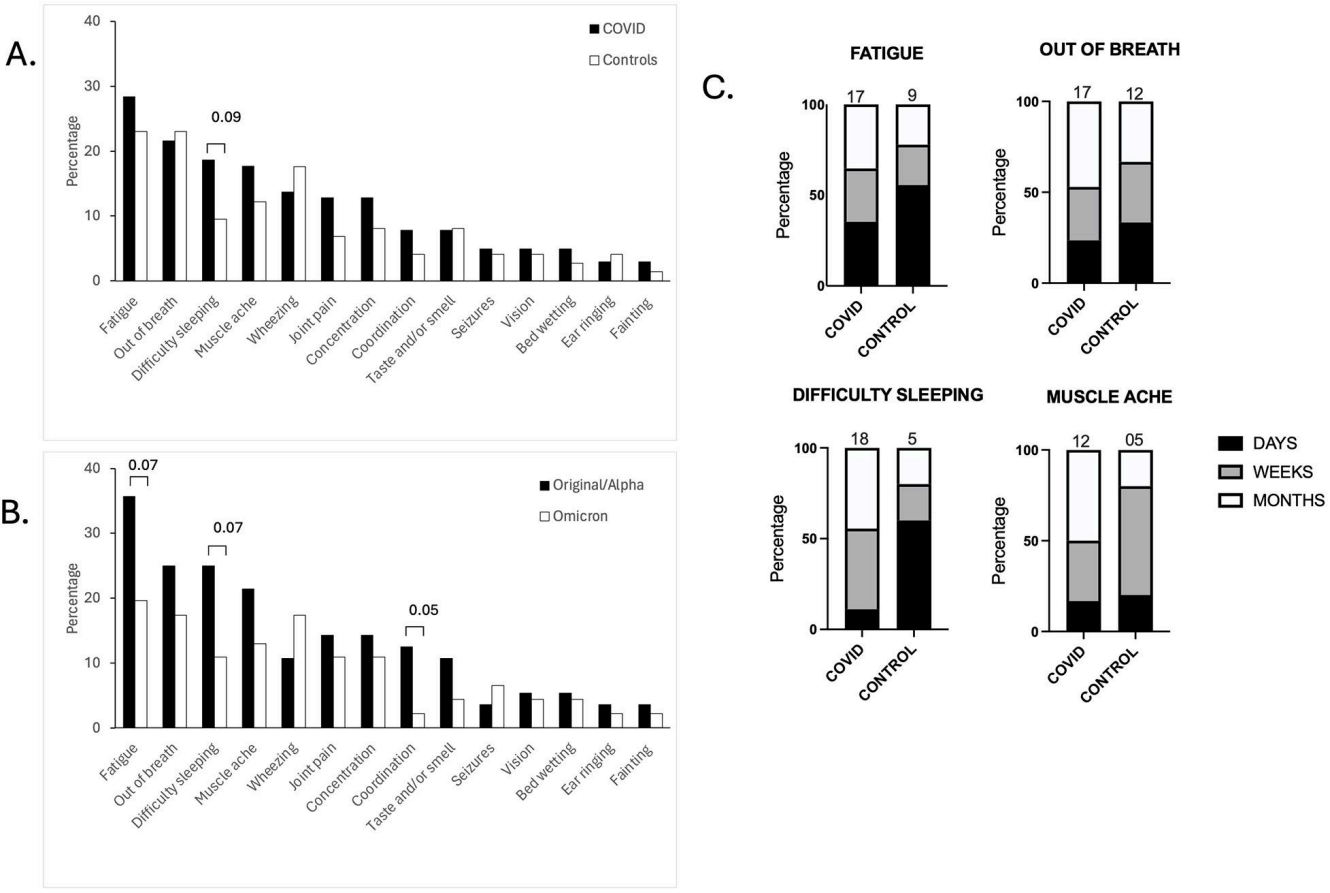
Posthospitalization symptoms by infection status and pandemic wave. **(A)** The percentage of COVID-positive and control patients reporting each symptom following hospitalization. A trend toward greater shortness of breath was observed in COVID-positive patients compared with controls (*p* = 0.09); no other significant differences were identified between the two groups. **(B)** The percentage of symptoms reported by pandemic wave (Original/Alpha vs. Omicron). Trends toward higher rates of fatigue (*p* = 0.07), difficulty sleeping (*p* = 0.07), and coordination difficulties (*p* = 0.05) were observed during the Original/Alpha wave than during the Omicron wave. **(C)** Duration of the four most commonly reported symptoms (fatigue, shortness of breath, difficulty sleeping, and muscle ache) in COVID-positive vs. control patients, expressed as the percentage of respondents reporting symptom duration of days, weeks, or months. The month category includes several months, many months, and greater than 1 year. The numbers above the columns indicate the total number of patients who responded to the duration question. There were no statistical differences between COVID and control patients. *p*-Values were calculated by using a chi-squared test.

The most common posthospitalization symptoms for children with COVID included fatigue, shortness of breath, and difficulty sleeping. These symptoms were similarly reported by uninfected controls, with no significant differences in symptom duration between groups (Figure 2). At least one symptom lasting several months or longer was reported by 13.7% of COVID-positive patients (14/102) and 10.8% of controls (8/74; *p* = 0.65). Among patients infected with the Original/Alpha variants (*n* = 56), fatigue (35.7%), shortness of breath (25.0%), and difficulty sleeping (25.0%) were the most reported symptoms. Among patients infected with the Omicron variant (*n* = 46), no individual symptom exceeded 20%, with fatigue (19.6%), wheezing (17.4%), and shortness of breath (17.4%) reported most frequently (Figure 2B). Fatigue, sleeping difficulties, and coordination problems all trended higher among patients infected with the Original/Alpha variants compared with those infected with Omicron (*p* = 0.07, 0.07, and 0.05, respectively).

### Psychometric testing

**Figure 3:**
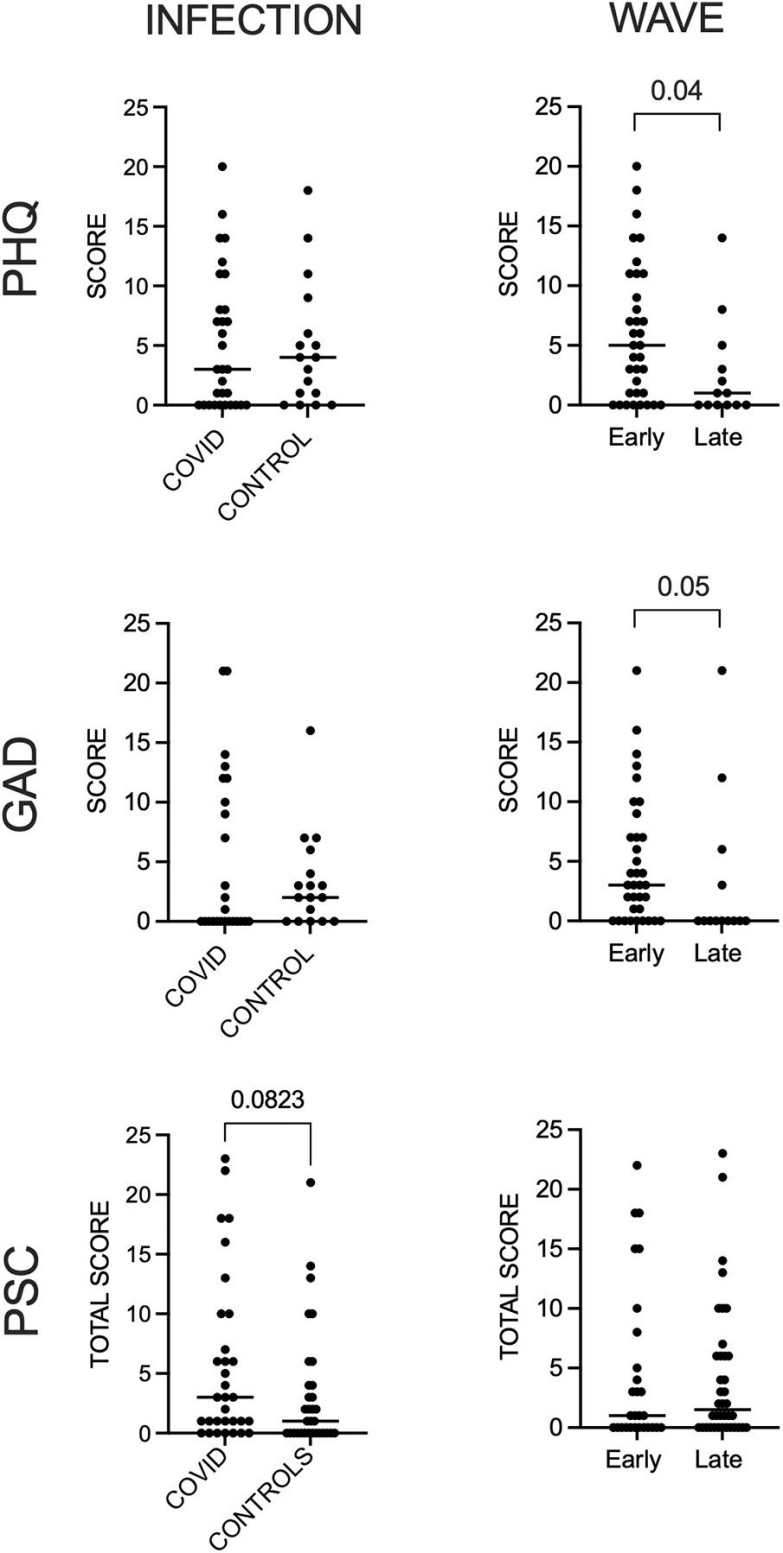
Modified PHQ-9, GAD-7, and PSC-17 scores by infection status and pandemic wave. Individual scores and median values are shown for each comparison. The left panels display scores stratified by infection status (COVID-positive vs. control). The right panels display scores stratified by pandemic wave, where Early refers to patients admitted during the Original/Alpha variant wave and Late refers to those admitted during the Omicron wave, independent of infection status. Higher PHQ-9 and GAD-7 scores indicate greater depression and anxiety symptom burden, respectively; higher PSC-17 total scores indicate greater behavioral and emotional difficulties. *p*-Values indicating statistical significance or trend are shown above brackets. Comparisons were performed using the Mann–Whitney *U*-test.

Psychometric scores and PSC-17 subscale results are shown individually and in aggregate in Figure 3 and Table 3, respectively. Modified PHQ-9 scores were available for 48 children in total. Overall, mild (score 5–10), moderate (score 11–14), moderately severe (score 15–19), and severe depression (score 20–24) were present in 10 (20.8%), 7 (14.6%), 2 (4.2%), and 2 (4.2%) patients, respectively. Modified PHQ-9, GAD-7, and PSC-17 total scores did not differ significantly between COVID-hospitalized patients and controls (*p* = 0.95, 0.73, and 0.08, respectively), with small effect sizes across all three measures (*r* = 0.05, 0.31, and 0.39) and confidence intervals crossing zero in each case. In addition, PSC-17 subscale positivity rates for Attention, Internalization, and Externalization were similarly comparable between the two groups.

**Table 3:** PSC-17 subscores and psychometric scores.

(A)								
	*n*	Cases median (IQR)	*n*	Controls median (IQR)	*p*-Value	*r*	95% CI	Power
PHQ-9	31	3 (0–8)	17	4 (1–6)	0.95	0.05	−0.53–0.63	0.07
GAD-7	31	3 (0–10)	17	2 (0–4)	0.73	0.31	−0.21–0.84	0.20
PSC-17 total	32	3 (1–9)	32	1 (0–4)	0.08	0.39	−0.12–0.90	0.42
Internalization	32	3 (9.4%)	32	1 (3.1%)	ns			
Externalization	32	3 (9.4%)	32	0 (0%)	ns			

(B)								
	*n*	Early (Alpha) median (IQR)	*n*	Late (Omicron) median (IQR)	*p*-Value	*r*	95% CI	Power
PHQ-9	35	5 (1–11)	13	1 (0–3)	0.04*	0.62	0.05–1.19	0.54
GAD-7	35	3 (0–7)	13	0 (0–3)	0.05	0.29	−0.47–1.05	0.20
PSC-17 total	25	1 (0–4)	39	2 (0–6)	0.27	0.09	−0.48–0.66	0.09
Attention	25	3 (12.0%)	39	3 (7.7%)	ns			
Internalization	25	2 (8.0%)	39	2 (5.1%)	ns			
Externalization	25	2 (8.0%)	39	1 (2.6%)	ns			

Scores are shown based on infection status (A) or phase of the pandemic (B). PHQ-9 and GAD-7 are shown as median (IQR). The PSC-17 subscale results show numbers of children with a positive subscore (defined as ≥7, ≥5, and ≥7 for Attention, Internalization, and Externalization scales, respectively), with proportions given in parentheses. Continuous measures were compared using the Mann–Whitney *U* test; subscore proportions were compared using the chi-squared or Fisher's exact test (ns, not significant). *r* refers to Rank-Biserial Correlation. Effect sizes are reported with 95% confidence intervals. Power reflects *post hoc* statistical power estimated by asymptotic relative efficiency.

* *p* value less 0.05.

When patients were stratified by pandemic era, modified PHQ-9 scores were significantly higher among those hospitalized during the Original/Alpha period compared with the Omicron period, regardless of infection status [median 5 (IQR 1–11) vs. 1 (IQR 0–3); *p* = 0.04; *r* = 0.62, 95% CI 0.05–1.19, Figure 3A]. This represents a moderate effect size, suggesting a clinically meaningful difference in depressive symptom burden between the two eras. GAD-7 scores trended in the same direction but did not reach statistical significance [median 3 (IQR 0–7) vs. 0 (IQR 0–3); *p* = 0.05; *r* = 0.29, 95% CI −0.47–1.05, Figure 3], reflecting a small-to-moderate effect. PSC-17 total scores did not differ significantly between the two eras (*p* = 0.27; *r* = 0.09, 95% CI −0.48–0.66), nor did subscale positivity rates for Attention, Internalization, or Externalization.

Bivariate analyses examining potential confounders of psychometric outcomes revealed no statistically significant associations between Modified PHQ-9, GAD-7, or PSC-17 scores and sex, age, ICU admission, MIS-C status, or length of stay (Supplementary Table 1).

### COVID-associated stressors

**Figure 4:**
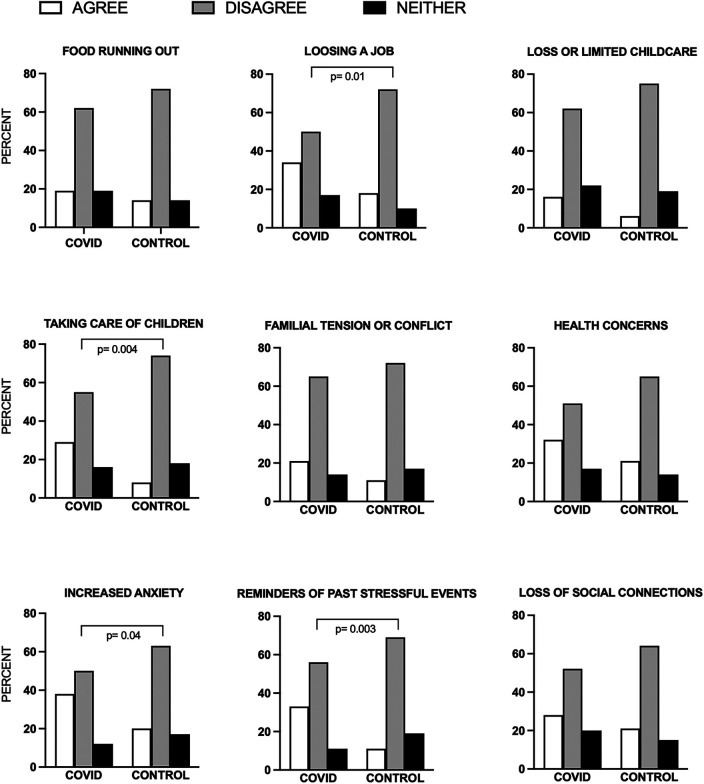
The proportion of COVID-positive patients and controls reporting COVID-related stress following hospitalization. Responses to nine stress domains are shown as percentages of patients agreeing (white bars), disagreeing (gray bars), or neither agreeing nor disagreeing (black bars) with each concern. COVID-positive families reported significantly greater stress related to losing a job (*p* = 0.01), childcare (*p* = 0.004), increased anxiety (*p* = 0.04), and reminders of past stressful events (*p* = 0.003). No significant differences were observed for the remaining domains. *p*-Values were calculated by using a chi-squared test and are shown above brackets.

Families of children infected with SARS-CoV-2 (*n* = 102) reported COVID-associated stress across multiple domains (Figure 4). More than 15% of families endorsed COVID-related stress in all queried areas, including food shortage, job loss, loss of housing or utilities, loss of childcare, taking care of children, family conflict, physical health concerns, increased anxiety, reminders of past stressful events, and loss of social connections. Families of hospitalized COVID-uninfected controls (*n* = 74) reported stress across similar domains but to a significantly lesser degree. Specifically, families of children with COVID reported significantly greater stress related to job loss (*p* = 0.01), taking care of children (*p* = 0.004), increased anxiety (*p* = 0.04), and reminders of past stressful events (*p* = 0.003) compared with controls. No significant differences in COVID-related stress were noted based on strain variant.

### Subsequent medical encounters

There were no differences in the reported rates of subsequent hospitalization between children with COVID (31/101, 30.7%) and controls (18/75, 24.3%, *p* = 0.4). There was also no difference in the reported proportion of children requiring ED or urgent care visits after admission. However, controls reported a higher median number of subsequent ED/urgent care visits compared with infected patients (3 vs. 2, *p* = 0.006) over the survey period. There were no strain-related differences in the proportion of children seeking care at an ED/urgent care after their COVID diagnosis (48.2% vs. 52.2% for Original/Alpha and Omicron strains, respectively, *p* = 0.7). However, children infected with Omicron were more likely to report subsequent hospitalization compared with those infected with the Original/Alpha variants (41.3% vs. 21.8%, *p* = 0.03). Children infected with Omicron were more likely to subsequently present with fever (37.0% vs. 19.6%, *p* = 0.05) and dehydration (13% vs. 1.8%, *p* = 0.03) compared with those with the Original/Alpha variants.

## Conclusions

A variety of persistent, adverse physical and mental effects in children have been attributed to the COVID pandemic. The syndrome of long COVID, similar to other chronic postinfectious syndromes such as chronic Lyme or EBV, is challenging to study. This difficulty reflects the variability in symptomatology, the absence of a specific diagnostic test, and an incomplete understanding of the underlying mechanism(s). In our study sample of one of the initial pediatric populations in the United States affected by COVID, 22% of children hospitalized with COVID required several months or more to recover, which is similar to what has been observed in some pediatric studies in association with COVID hospitalization (30). When examined at the level of individual symptoms rather than overall recovery, 14/102 (13.7%) of children with COVID reported at least one individual symptom lasting several months or longer, broadly consistent with the global recovery estimate.

There were significant strain-related differences in the time to recovery following hospitalization for COVID, with almost a third of patients hospitalized with the Original/Alpha variant and less than 10% of patients hospitalized with the Omicron variant reporting time to full recovery of several months or more. These findings are consistent with those of studies done primarily in adults but also in some children showing a lower incidence of long COVID with Omicron infection (31–33). The basis of this strain-related difference in long COVID incidence and severity has been hypothesized to result from differences in acute disease severity, which is consistent with our findings of increased length of hospitalization stay and greater ICU admission rates in the first wave. In addition, patients infected with the Original/Alpha variant were older, another factor that has been identified as a risk factor for both acute and long COVID in children (33). The decreased severity of acute COVID (which, in turn, may result in a decreased risk for long COVID) in the latter waves has been attributed to a variety of factors. These include mutations in the spike protein that resulted in decreased lower respiratory tract viral replication and decreased ability to inhibit host interferon responses. The development of immunologic memory from exposure to the early variants and, in some cases, vaccines, may have also contributed to reduced severity observed in this and other studies comparing Omicron and the Original/Alpha variant. Specifically, the rates of MIS-C have declined significantly.

The most common symptoms experienced by children in our study after COVID hospitalization were, in decreasing order, fatigue, feeling out of breath, difficulty sleeping, and muscle aches. However, these symptoms were also common among controls. Previous studies have shown a similar pattern of symptomatology among children with long COVID (30, 34), with some recent studies suggesting that the pattern of symptoms may vary with age (35). Notably, the type and duration of individual symptoms did not differ significantly between hospitalized children. This finding underscores the importance of including contemporaneous control groups in studies of postacute COVID sequelae, as symptoms such as fatigue and sleep disturbance appear common among hospitalized children regardless of infection status.

The COVID pandemic has been associated with higher rates of mental illness in children (36). Loss of routine and social support systems, including school closures, social isolation, uncertainty regarding disease severity, caregiver stress, and economic instability, have been implicated in this process (37–40). Evidence for these effects is substantial. In a systematic review involving 80,000 children, school closures during the original COVID wave were associated with emotional, behavioral, and restlessness/inattention problems, with 18%–60% of children scoring above risk thresholds for psychological distress, particularly anxiety and depression (41). In addition to the effects of societal disruption, direct effects of SARS-CoV-2 infection on mental health have been hypothesized, through cytokine dysregulation, effects on the vascular endothelium, and alterations in residential cell phenotype [reviewed in (42)].

Overall, 43.8% of our cohort had at least mild depression by PHQ-9 screening and 20.9% had at least moderate depression. These prevalence rates are clinically concerning, independent of between-group statistical comparisons, but are comparable with other reports. In one large meta-analysis involving 80,879 youths, the pooled prevalence rates of clinically elevated depression and anxiety during the COVID-19 pandemic were 25.2% and 20.5%, respectively (10). Limited data are available regarding the burden of mental illness across pandemic waves, although several studies have reported persistently high rates during the Omicron period (43, 44).

No significant differences in psychometric scores were identified based on COVID infection status among hospitalized children. However, modified PHQ-9 scores were significantly higher among children hospitalized during the Original/Alpha wave than among those hospitalized during the Omicron wave, and GAD-7 scores trended in the same direction, although this did not reach statistical significance (*p* = 0.05). Although societal disruption was not directly measured in this study, we note that in contrast to the Original/Alpha wave—during which New York City public schools were fully closed for in-person learning for an extended period—in-person schooling was maintained as an explicit policy priority during the Omicron wave despite surging case counts (45). Together with the broader normalization of daily routines by the later pandemic period, our findings are consistent with the hypothesis that societal disruption, rather than SARS-CoV-2 infection *per se*, played an important role in the psychiatric symptoms observed in our cohort. Given the modest sample size of this study, null findings in between-group comparisons should be interpreted cautiously, as true differences may have gone undetected.

We did not find a significant difference in PSC-17 scores based on the COVID-19 wave. This, however, may reflect a lower sensitivity of the total global score to the specific emotional effects of the pandemic. The total score merges internalizing, externalizing, and attention domains, and opposing trends between these subscales may mask domain-specific changes. The accuracy of these results may have also been influenced by parental stress, which fluctuated significantly during the different phases of the pandemic (46, 47).

Our findings must be studied within the context of the relatively high rates of mental health issues in children from the Bronx. Although prepandemic mental health data precisely matching the methods utilized for this study are not available, a pre-COVID study of children attending an asthma center indicated that 21.6% of patients demonstrated clinically significant symptoms of anxiety and/or depression (48). Studies of children and adolescents living in the Bronx and Harlem indicated that depression rates were most heavily correlated with children's perceptions of their neighborhood quality (49). Collectively, these findings suggest that the children in our study may carry a heightened vulnerability to the psychiatric consequences of the COVID-19 pandemic.

Families reported relatively high levels of COVID-related stress following hospitalization, independent of COVID status. This observation is consistent with the high levels of poverty in the Bronx, with 27.7% of the population living below the poverty level and approximately 39% experiencing food insecurity (50, 51). Importantly, when compared with controls, families of children hospitalized with COVID reported significantly high levels of COVID-related stress around several areas, including losing a job, taking care of children, anxiety, and reminders of past stressful events. Hospitalization for COVID can impose a range of psychosocial stressors on families, which can be exacerbated by poverty (52). Stress has been associated with worse physical and mental health outcomes following COVID in several studies. In a prospective study of 790 adults hospitalized for COVID, life stressors were associated with impaired performance of activities of daily living, worse mental health outcomes, and prolonged symptoms (53). Furthermore, the presence of stress prior to COVID has been associated with the development of persistent COVID symptoms (54, 55).

No differences in subsequent hospitalizations were noted between controls and children hospitalized with COVID. However, we did detect an increased number of ED/urgent care visits in control patients compared with children infected with COVID, although the basis for this is unclear. It is possible that after being hospitalized for COVID, patients and families were more reluctant to engage the healthcare system than controls. Reluctance to seek healthcare during the original COVID outbreak because of fear of contracting COVID was well documented (56). We found that children hospitalized with Omicron infections were more likely to report subsequent hospitalizations and symptoms of fever and dehydration when compared with those infected with the Original/Alpha variants. We suspect that at least a significant component of this difference may be due to the younger age of children hospitalized with Omicron compared with the Original/Alpha variants.

This study has several important limitations. The small overall sample size and the limited number of psychiatric assessments reduce statistical power and preclude definitive conclusions regarding associations between infection status, pandemic phase, and psychiatric outcomes. In addition, the retrospective nature of the study imparts a substantial risk of recall bias. Additional limitations include the absence of prehospitalization baseline psychiatric assessments, which precludes determination of whether observed symptoms represent new-onset or pre-existing psychopathology. The heterogeneity of the control group patients, who were hospitalized for a variety of non-COVID conditions, may also limit comparability. In addition, patients were matched for the presence of chronic disease but not severity and this could serve as a source of potential residual confounding. The single-center design limits generalizability to other pediatric populations. Furthermore, there was a differential response rate between Original/Alpha controls and Omicron controls (25.6% vs. 37.3%, *p* = 0.047), which introduces the possibility of selection bias in wave-based comparisons.

In conclusion, we found that one in five children hospitalized with COVID reported that their disease took months to resolve. Notably, patients hospitalized with the Original/Alpha variant were significantly more likely to report a prolonged recovery compared with those hospitalized during the Omicron wave. Interestingly, children with COVID and controls reported similar posthospitalization symptoms and duration of symptoms, underscoring the importance of including a contemporaneous control group in studies of postacute COVID sequelae. An increase in depression and anxiety scores was not observed in association with infection status but was observed during the earlier phase of the pandemic, independent of infection. These findings highlight the potential contribution of societal disruption during the early COVID-19 pandemic to the mental health of children, as opposed to the effects of the infection itself. Children affected by the COVID pandemic in the Bronx represent the leading edge of long-term epidemiologic trends, and these observations enhance our understanding of the long-term impact of COVID in children.

## Data Availability

The raw data supporting the conclusions of this article will be made available by the authors, without undue reservation.
