# Supplementary material for: Persistent physical and mental health effects of COVID-19 hospitalization
Source: Front Pediatr. 2026 Jul 30;14:1710900. doi: 10.3389/fped.2026.1710900 (PMC13469453; doi:10.3389/fped.2026.1710900)
Supplement: Supplementary file 1 [file Datasheet1.pdf]

Long-term COVID-19 Follow up in Pediatric Pts (Parent survey)

Please complete the survey below.

Complete el siguiente cuestionario.

SECTION 1: Medical Care

According to our records, your child had been admitted to the hospital on [hosp_date_control], and during this hospitalization they had tested positive for COVID.

The following survey questions will be referring to this specific hospitalization.

Has your child ever been diagnosed with COVID-19?<br><br>¿Alguna vez su hijo ha sido diagnosticado con COVID-19?	◯ Yes<br>◯ No
When was your child first diagnosed with COVID-19? (best estimate)<br><br>¿Cuándo fue su hijo diagnosticado con COVID-19? (mejor estimado)	__________________________________
Was your child ever hospitalized with a diagnosis of COVID? (includes testing positive for COVID while hospitalized for a different reason)<br><br>¿Alguna vez ha sido su hijo hospitalizado con el diagnóstico de COVID? (se incluye dar positivo por COVID mientras hospitalizado por otra razón)	◯ Yes<br>◯ No
Has your child had any Emergency Department or Urgent Care visits over the last 2 years? (since February 2020)<br><br>¿Ha tenido su hijo alguna visita al Departamento de Emergencias o al Cuarto de Urgencias en los últimos 2 años? (desde Febrero 2020)	◯ Yes<br>◯ No
How many times?<br><br>¿Cuántas veces?	__________________________________
Has your child had any Emergency Department or Urgent Care visits (for any reason, including non-COVID reasons) since the initial hospitalization with COVID-19 on [hosp_date_control]?<br><br>¿Ha tenido su hijo alguna visita al Departamento de Emergencias o al Cuarto de Urgencias (por cualquier razón, incluyendo razones no relacionadas a COVID) desde su primera hospitalización por COVID-19 en [hosp_date_control]?	◯ Yes<br>◯ No

How many times?

¿Cuántas veces?

____

Please check off reasons for visit to the Emergency Department or Urgent Care. (may check more than one box)

Por favor seleccione las razones de las visitas al Departamento de Emergencia o al Cuarto de Urgencias. (puede seleccionar más de una opción)

- ☐ Breathing problems (for example, shortness of breath, cough, breathing fast, wheezing) -- Problemas para respirar (por ejemplo falta de aire, tos, respiración rápida, sibilancias)
- ☐ Stomach problems (for example nausea, vomiting, diarrhea, stomach pains) -- Problemas estomacales (por ejemplo náuseas, vómitos, diarrea, dolor estomacal)
- ☐ Fever -- Fiebre
- ☐ Blood clot -- Coágulo en la sangre
- ☐ Dehydration -- Deshidratación
- ☐ Behavioral (for example, depression, anxiety, acting out, bed wetting) -- Problemas de comportamiento (por ejemplo, depresión, ansiedad, mal comportamiento, mojar la cama)
- ☐ Neurologic (for example, seizures) -- Problemas neurológicos (por ejemplo, convulsiones)
- ☐ General (for example, weakness, weight loss) -- General (por ejemplo, debilidad, pérdida de peso)
- ☐ Visual problems -- Problemas visuales
- ☐ Smelling problems -- Problemas con olores
- ☐ Other -- Otro

If other, please specify.

Si su respuesta es otro, por favor especificar

____

Was your child ever hospitalized for a reason other than COVID-19 over the last 2 years? (since February 2020)

¿Alguna vez su hijo ha sido hospitalizado por otra razón que no sea COVID-19 en los últimos 2 años? (desde Febrero 2020)

- ◯ Yes
- ◯ No

How many times?

¿Cuántas veces?

____

Has your child had any hospitalizations (for any reason, including non-COVID reasons) since the initial hospitalization with COVID-19 on [hosp_date_control]?

¿Alguna vez su hijo ha sido hospitalizado (por cualquier razón, incluyendo razones no relacionadas a COVID) desde su primera hospitalización por COVID-19 en [hosp_date_control]?

- ◯ Yes
- ◯ No

How many times has your child been hospitalized since their initial hospitalization with COVID-19?

¿Cuántas veces su hijo ha sido hospitalizado desde su primera hospitalización por COVID-19?

____

Please check reasons for hospitalization since your child's initial hospitalization for COVID-19 on [hosp_date_control] (may check more than one box)<br><br>Por favor seleccione las razones de hospitalización desde la primera hospitalización por COVID-19 de su hijo en [hosp_date_control]. (puede seleccionar más de una opción)	☐ Breathing problems (for example, shortness of breath, cough, breathing fast) -- Problemas para respirar (por ejemplo falta de aire,tos, respiración rápida, sibilancias)<br>☐ Stomach problems (for example nausea, vomiting, diarrhea, stomach pains) -- Problemas estomacales (por ejemplo náuseas, vómitos, diarrea, dolor estomacal)<br>☐ Fever -- Fiebre<br>☐ Blood clot -- Coágulo en la sangre<br>☐ Dehydration -- Deshidratación<br>☐ Behavioral problems (for example depression with thoughts of suicide) -- Problemas de comportamiento (por ejemplo, depresión con pensamiento de suicidio)<br>☐ Neurologic (for example seizures) -- Problemas neurológicos (por ejemplo, convulsiones)<br>☐ Other -- Otro
If other, please specify.<br><br>Si su respuesta es otro, por favor especificar	__________________________________

SECTION 2: COVID Symptoms - Current or past (child < 16 years old)

SECCIÓN 2: Síntomas de COVID - Actual o pasado (niños < 16 años de edad)

Has your child completely recovered from COVID?<br><br>¿Se ha recuperado su hijo por completo del COVID?	◯ Yes -- Sí<br>◯ No<br>◯ My child did not have any symptoms of COVID -- Mi hijo no tenía ningún síntoma de COVID
If yes, how long did it take your child to completely recover?<br><br>Si su respuesta es sí, ¿cuánto tiempo le tomó a su hijo recuperarse totalmente?	◯ Days (1-7) -- Días (1-7)<br>◯ Weeks -- Semanas<br>◯ Several Months (for example 1-6 months) -- Varios meses (por ejemplo 1-6 meses)<br>◯ Many months (6 months - 1year) -- Muchos meses (6 meses - 1 año)<br>◯ More than 1 year -- Más de 1 año<br>◯ Other -- Otro
If other, please specify how long it took to recover.<br><br>Si su respuesta es otro, por favor especificar el tiempo que le tomó en recuperarse	__________________________________
If your child has not recovered, please check off how long the symptoms have been present?<br><br>Si su hijo no se ha recuperado, por favor seleccione ¿cuánto tiempo los síntomas han estado presentes?	◯ Days (1-7) -- Días (1-7)<br>◯ Weeks -- Semanas<br>◯ Several Months (for example 1-6 months) -- Varios meses (por ejemplo 1-6 meses)<br>◯ Many months (6 months - 1year) -- Muchos meses (6 meses - 1 año)<br>◯ More than 1 year -- Más de 1 año<br>◯ Other -- Otro

Did your child experience any of the problems below after being discharged from their hospitalization on [hosp_date_control]? Please check off yes/no and if yes give the time of symptoms.

¿Ha experimentado su hijo alguno de los síntomas mencionados abajo después de su salida del hospital en [hosp_date_control]? Por favor escoja sí/no y si su respuesta es sí mencione el tiempo de los síntomas.

	No	Yes (If yes then also select a time of symptoms) -- Sí (Si su respuesta es sí seleccione el tiempo de los síntomas tambien)	Days (1-7 days) -- Dias (1-7 dias)	Weeks (More than 1 week) -- Semanas(Mas de 1 semana)	Several Months (More than 1 month) -- Varios Meses (Mas de 1 mes)	Many months (More than 6 months) -- Muchos meses (Mas de 6 meses)	More than 1 year -- Mas de 1año
Seizures -- Convulsiones	☐	☐	☐	☐	☐	☐	☐
Ringing in your ears -- Zumbido en los oídos	☐	☐	☐	☐	☐	☐	☐
Feeling out of breath or difficulty breathing -- Sensación de falta de aire o dificultad para respirar	☐	☐	☐	☐	☐	☐	☐
Wheezing (high pitched whistling sound made while breathing) -- Sibilancias	☐	☐	☐	☐	☐	☐	☐
Joint pain -- Dolor en las articulaciones	☐	☐	☐	☐	☐	☐	☐
Muscle aches -- Dolor muscular	☐	☐	☐	☐	☐	☐	☐
Fatigue/tiredness -- Fatiga/cansancio	☐	☐	☐	☐	☐	☐	☐
Problems concentrating -- Problemas de concentración	☐	☐	☐	☐	☐	☐	☐
Vision problems -- Problemas de visión	☐	☐	☐	☐	☐	☐	☐
Poor coordination (ex: Change in balance) -- Poca coordinación (ejemplo: cambios en el balance)	☐	☐	☐	☐	☐	☐	☐

Changes in taste or smell -- Cambios en sabor u olor	☐	☐	☐	☐	☐	☐	☐
Difficulty sleeping or change in sleep patterns since COVID-19 diagnosis -- Dificultad para dormir o cambio en el patrón de sueño desde su diagnóstico de COVID-19	☐	☐	☐	☐	☐	☐	☐
Bedwetting -- Incontinencia nocturna	☐	☐	☐	☐	☐	☐	☐
Fainting -- Desmayos	☐	☐	☐	☐	☐	☐	☐
Other -- Otro	☐	☐	☐	☐	☐	☐	☐

If other, please specify.

Si su respuesta es otro, por favor especificar. ________________________

For the following symptoms that your child experienced after being discharged from their hospitalization on [hosp_date_control], was the symptom a new symptom? Or was it a worsening of a symptom that existed before this specific hospitalization?

De los siguientes síntomas que su hijo ha experimentado después de que le dieran salida de su hospitalización en [hosp_date_control], es este síntoma un nuevo síntoma? O es un síntoma que ya estaba presente antes de este hospitalización y que luego empeoró?

	New symptom since being discharged from hospitalization on [date_admit] (Nuevo síntoma desde que le dieron salida de su hospitalización en [date_admit])	Worsening of a preexisting symptom that started before your hospitalization on [date_admit] (Síntoma que empezó antes de su hospitalización en [date_admit] y que luego empeoró)
Seizures -- Convulsiones	○	○
Ringing in your ears -- Zumbido en los oídos	○	○
Feeling out of breath or difficulty breathing -- Sensación de falta de aire o dificultad para respirar	○	○
Wheezing (high pitched whistling sound made while breathing) -- Sibilancias	○	○
Joint pain -- Dolor en las articulaciones	○	○
Muscle aches -- Dolor muscular	○	○

Fatigue/tiredness -- Fatiga/cansancio	○	○
Problems concentrating -- Problemas de concentración	○	○
Vision problems -- Problemas de visión	○	○
Poor coordination (ex: Change in balance) -- Poca coordinación (ejemplo: cambios en el balance)	○	○
Changes in taste or smell -- Cambios en sabor u olor	○	○
Difficulty sleeping or change in sleep patterns since COVID-19 diagnosis -- Dificultad para dormir o cambio en el patrón de sueño desde su diagnóstico de COVID-19	○	○
Bedwetting -- Incontinencia nocturna	○	○
Fainting -- Desmayos	○	○
Other -- Otro	○	○

SECTION 3: Social and Familial History
SECCIÓN 3: Historia Social y Familiar

The current coronavirus (COVID-19) is causing extra stress for many people, including families with children of all ages. We would like to know how things are going for you and your family related to this situation. Please answer the following questions about your experiences and feelings over the last few weeks.
El presente coronavirus (COVID-19) le está causando a muchas personas estrés adicional, incluyendo familias con niños de todas las edades. Nos gustaría poder conocer cómo las cosas han estado para usted y su familia en relación a esta situación. Por favor contestar las siguientes preguntas a cerca de su experiencia y sentimientos en las últimas semanas

Because of COVID-19 related events and changes, I have felt increased stress about:
En relación a los eventos y cambios del COVID-19, me he sentido estresado a cerca de:

	Strongly Disagree -- Muy en desacuerdo	Somewhat Disagree -- Algo en desacuerdo	Neither Agree nor Disagree -- Ni de acuerdo ni en desacuerdo	Somewhat Agree -- Algo en acuerdo	Strongly Agree -- Muy en acuerdo

Food running out or being unavailable -- La comida se acaba o no está disponible	○	○	○	○	○
Losing a job or decrease in family income -- Perder el trabajo o disminución del ingreso familiar	○	○	○	○	○
Housing or utilities -- Vivienda o servicios públicos	○	○	○	○	○
Loss of or limited childcare -- Pérdida o el cuidado limitado de los niños	○	○	○	○	○
Taking care of children, including those who are normally in school -- Cuidar a los niños incluyendo a los que van a la escuela	○	○	○	○	○
Tension or conflict between household members-- Tensión o conflicto entre los miembros de la familia	○	○	○	○	○
Physical health concerns for me or a family member -- Preocupación acerca de mi salud física o de un miembro de la familia	○	○	○	○	○
Increased anxiety or depression -- Aumento en la ansiedad o depresión	○	○	○	○	○
Reminders of past stressful/traumatic events -- Recuerdos de situaciones estresantes en el pasado/eventos traumáticos	○	○	○	○	○
Loss of social connections, social isolation -- Pérdida de conexión social, aislamiento social	○	○	○	○	○
Increased tension around school work at home -- Aumento de tensión sobre el trabajo escolar en la casa	○	○	○	○	○

SECTION 6: BEHAVIOR SCREEN FOR CHILD
(Completed by Parents/Caregivers of patients 4-11 years old)
SECCIÓN 6: EVALUACIÓN DEL COMPORTAMIENTO PARA EL NIÑO
(Completar por el Padre/Cuidador del paciente de 4-11 años de edad)

Please read each item carefully and mark the response that best describes your child.
Por favor leer cuidadosamente cada elemento y seleccionar la respuesta que mejor describa a su niño.

	0-- Never (Nunca)	1-- Sometimes (Algunas veces)	2-- Often (A menudo)
Fidgety, unable to sit still -- Nervioso, incapaz de quedarse sentado quieto	◯	◯	◯
Feels sad, unhappy -- Triste, infeliz	◯	◯	◯
Daydreams too much -- Sueña despierto demasiado	◯	◯	◯
Refuses to share -- Se rehúsa a compartir	◯	◯	◯
Does not understand other people's feelings -- No entiende los sentimientos de los demás	◯	◯	◯
Feels hopeless -- Se siente sin esperanzas	◯	◯	◯
Has trouble concentrating -- Problemas para concentrarse	◯	◯	◯
Fights with other children -- Pelea con otros niños	◯	◯	◯
Is down on himself or herself -- Duro consigo mismo	◯	◯	◯
Blames others for his or her troubles -- Culpa a otros por sus problemas	◯	◯	◯
Seem to be having less fun -- Parece tener menos diversión	◯	◯	◯
Does not listen to rules -- No escucha las reglas	◯	◯	◯
Acts as if driven by a motor -- Actúa como impulsado por un motor	◯	◯	◯
Teases others -- Molesta a otros	◯	◯	◯
Worries a lot -- Se preocupa demasiado	◯	◯	◯

Takes things that do not belong to him or her -- Toma cosas que no le pertenecen	○	○	○
Distracted easily -- Se distrae fácilmente	○	○	○

Internalizing Score ______________________

Score: ______________________

SECTION 7 (for parents)
SECCIÓN 7 (para los padres)

In the last year have you (or your child) seen your primary care provider, either via telehealth or in person?

En el último año usted (o su hijo) ha visto a su proveedor de atención primaria, ya sea por vía telemedicina o en persona?

- ○ Yes
- ○ No

If no, why not? (may check more than one)

Si su respuesta es no, ¿por qué no? (puede seleccionar más de una)

- ☐ Wasn't told it was needed -- No me dijeron que era necesario
- ☐ Worried about getting COVID -- Me preocupaba obtener COVID
- ☐ Could not get an appointment -- No pude conseguir cita
- ☐ Too busy -- Demasiado ocupado
- ☐ Didn't feel it was necessary. -- Sentí que no era necesario
- ☐ Other -- Otro

If other, please specify.

Si su respuesta es otro, por favor especificar ______________________

If yes, how many times?

Si su respuesta es sí, ¿cuántas veces? ______________________

Have you received the SARS-CoV-2 vaccine?

¿Ha recibido la vacuna para SARS-COV-2?

- ○ Yes
- ○ No

If no, please check off the reason why. (may check more than one)

Si su respuesta es no, por favor seleccionar la razón (puede seleccionar más de una)

- ☐ Don't feel it is needed -- Sentí que no era necesario
- ☐ Concerned about side effects -- Me preocupaba acerca de los efectos secundarios
- ☐ Too much work required -- Requería demasiado trabajo
- ☐ Tried to, but was unable to get -- Lo intenté, pero no lo pude obtener
- ☐ Other -- Otro

If other, please specify.<br><br>Si su respuesta es otro, por favor especificar	__________________________________
Is your child eligible to receive the COVID vaccine?<br><br>¿Es su hijo elegible para recibir la vacuna de COVID?	◯ Yes -- Sí<br>◯ No<br>◯ I don't know -- No lo sé
If yes, has your child received or been scheduled to receive the vaccine?<br><br>Si su respuesta es sí, ¿Ha recibido su hijo o ha sido programado para recibir la vacuna?	◯ Yes<br>◯ No
If no, please check off the reason why. (may check more than one)<br><br>Si su respuesta es no, por favor seleccionar la razón (puede seleccionar más de una)	☐ Don't feel it is needed -- Sentí que no era necesario<br>☐ Concerned about side effects -- Me preocupaba acerca de los efectos secundarios<br>☐ Too much work required -- Requería demasiado trabajo<br>☐ Tried to, but was unable to get -- Lo intenté, pero no lo pude obtener<br>☐ Other -- Otro
If other, please specify.<br><br>Si su respuesta es otro, por favor especificar	__________________________________
Would you like to get a summary of the overall results from this study on the long-term effects of COVID in children?<br><br>¿Le gustaría recibir un resumen de los resultados de este estudio acerca de los efectos a largo plazo del COVID en niños?	◯ Yes<br>◯ No
Would you like to be included in a future study looking at effects of a child's COVID hospitalization on their parents?<br><br>¿ Te gustaría ser incluído en un estudio sobre los efectos en los padres de la hospitalización de un niño con COVID?	◯ Yes<br>◯ No
