# Supplementary material for: Persistent physical and mental health effects of COVID-19 hospitalization
Source: Front Pediatr. 2026 Jul 30;14:1710900. doi: 10.3389/fped.2026.1710900 (PMC13469453; doi:10.3389/fped.2026.1710900)
Supplement: Supplementary file 2 [file Datasheet2.pdf]

Long-term COVID-19 Follow up in Pediatric Pts (Patient survey)

Please complete the survey below.

Complete el siguiente cuestionario.

SECTION 1: Medical Care

According to our records, you had been admitted to the hospital on [hosp_date_control], and during this hospitalization you had tested positive for COVID.

The following survey questions will be referring to this specific hospitalization.

Have you ever been diagnosed with COVID-19?<br><br>¿Alguna vez has sido diagnosticado con COVID-19?	◯ Yes<br>◯ No
When were you first diagnosed with COVID-19? (best estimate)<br><br>¿Cuándo fuiste diagnosticado con COVID-19 por primera vez? (mejor estimado)	______________________________
Were you ever hospitalized with a diagnosis of COVID? (includes testing positive for COVID while hospitalized for a different reason)<br><br>¿Alguna vez has sido hospitalizado por un diagnóstico de COVID? (se incluye dar positivo por COVID mientras hospitalizado por otra razón)	◯ Yes<br>◯ No
Have you had any Emergency Department or Urgent Care visits over the last 18 months? (since February 2020)<br><br>¿Has tenido alguna visita al Departamento de Emergencias o al Cuarto de Urgencias en los últimos 18 meses? (desde Febrero 2020)	◯ Yes<br>◯ No
How many times?<br><br>¿Cuántas veces?	______________________________
Have you had any Emergency Department or Urgent Care visits (for any reason, including non-COVID reasons) since the initial hospitalization with COVID-19 on [hosp_date_control]?<br><br>¿Has tenido alguna visita al Departamento de Emergencias o al Cuarto de Urgencias (por cualquier razón, incluyendo razones no relacionadas a COVID) desde su primera hospitalización por COVID-19 en [hosp_date_control]?	◯ Yes<br>◯ No

How many times?

¿Cuántas veces?

____

Please check off reasons for visit to the Emergency Department or Urgent Care. (may check more than one box)

Por favor seleccione las razones de las visitas al Departamento de Emergencia o al Cuarto de Urgencias. (puede seleccionar más de una opción)

- ☐ Breathing problems (for example, shortness of breath, cough, breathing fast, wheezing) -- Problemas para respirar (por ejemplo falta de aire, tos, respiración rápida, sibilancias)
- ☐ Stomach problems (for example nausea, vomiting, diarrhea, stomach pains) -- Problemas estomacales (por ejemplo náuseas, vómitos, diarrea, dolor estomacal)
- ☐ Fever -- Fiebre
- ☐ Blood clot -- Coágulo en la sangre
- ☐ Dehydration -- Deshidratación
- ☐ Behavioral (for example, depression, anxiety, acting out, bed wetting) -- Problemas de comportamiento (por ejemplo, depresión, ansiedad, mal comportamiento, mojar la cama)
- ☐ Neurologic (for example, seizures) -- Problemas neurológicos (por ejemplo, convulsiones)
- ☐ General (for example, weakness, weight loss) -- General (por ejemplo, debilidad, pérdida de peso)
- ☐ Visual problems -- Problemas visuales
- ☐ Smelling problems -- Problemas con olores
- ☐ Other -- Otro

If other, please specify.

Si su respuesta es otro, por favor especificar

____

Were you ever hospitalized for a reason other than COVID-19 over the last 2 years? (since February 2020)

¿Alguna vez has sido hospitalizado por otra razón que no sea COVID-19 en los últimos 2 años? (desde Febrero 2020)

- ◯ Yes
- ◯ No

How many times?

¿Cuántas veces?

____

Have you had any hospitalizations (for any reason, including non-COVID reasons) since the initial hospitalization with COVID-19 on [hosp_date_control]?

¿Alguna vez has sido hospitalizado (por cualquier razón, incluyendo razones no relacionadas a COVID) desde su primera hospitalización por COVID-19 en [hosp_date_control]?

- ◯ Yes
- ◯ No

How many times have you been hospitalized since the initial hospitalization with COVID-19?

¿Cuántas veces has sido hospitalizado desde la primera hospitalización por COVID-19?

____

Please check reasons for hospitalization since your initial hospitalization for COVID-19 on [hosp_date_control] (may check more than one box)<br><br>Por favor seleccione las razones de hospitalización desde su primera hospitalización por COVID-19 en [hosp_date_control]. (puede seleccionar más de una opción)	☐ Breathing problems (for example, shortness of breath, cough, breathing fast) -- Problemas para respirar (por ejemplo falta de aire,tos, respiración rápida, sibilancias)<br>☐ Stomach problems (for example nausea, vomiting, diarrhea, stomach pains) -- Problemas estomacales (por ejemplo náuseas, vómitos, diarrea, dolor estomacal)<br>☐ Fever -- Fiebre<br>☐ Blood clot -- Coágulo en la sangre<br>☐ Dehydration -- Deshidratación<br>☐ Behavioral problems (for example depression with thoughts of suicide) -- Problemas de comportamiento (por ejemplo, depresión con pensamiento de suicidio)<br>☐ Neurologic (for example seizures) -- Problemas neurológicos (por ejemplo, convulsiones)<br>☐ Other -- Otro
If other, please specify.<br><br>Si su respuesta es otro, por favor especificar	__________________________________

SECTION 2: COVID Symptoms - Current or past (>16years old)
SECCIÓN 2: Síntomas de COVID- Actual o pasado (> 16 años de edad)

Have you completely recovered from COVID?<br><br>Se ha recuperado totalmente del COVID?	◯ Yes -- Sí<br>◯ No<br>◯ I did not have any symptoms of COVID --Yo no tenía ningún síntoma de COVID
If yes, how long did it take you to completely recover?<br><br>Si su respuesta es Sí, por cuánto tiempo le tomó en recuperarse?	◯ Days (1-7) -- Días (1-7)<br>◯ Weeks -- Semanas<br>◯ Several Months (for example 1-6 months) -- Varios Meses (por ejemplo 1-6 meses)<br>◯ Many months (6 months - 1year) -- Muchos meses (6 meses - 1 año)<br>◯ More than 1 year -- Más de 1 año<br>◯ Other -- Otro
If other, please specify how long it took to recover.<br><br>Si su respuesta fue otra, por favor especifique el tiempo que tomó en recuperarse	__________________________________
If you have not recovered, please check off how long the symptoms have been present?<br><br>Si no se ha recuperado, por favor escoja por cuánto tiempo los síntomas han estado presentes?	◯ Days (1-7) -- Días (1-7)<br>◯ Weeks -- Semanas<br>◯ Several Months (for example 1-6 months) -- Varios Meses (por ejemplo 1-6 meses)<br>◯ Many months (6 months - 1year) -- Muchos meses (6 meses - 1 año)<br>◯ More than 1 year -- Más de 1 año

Did you experience any of the problems below after discharge from your hospitalization on [hosp_date_control]? Please check off yes/no and if yes give the time of symptoms.

Ha experimentado alguno de los síntomas mencionados abajo después de su salida del hospital en [hosp_date_control]? Por favor escoja sí/no y si su respuesta es sí mencione el tiempo de los síntomas.

	No	Yes (If yes then also select a time of symptoms) -- Sí (Si su respuesta es sí seleccione el tiempo de los síntomas tambien)	Days (1-7 days) -- Dias (1-7 dias)	Weeks (More than 1 week) -- Semanas(Mas de 1 semana)	Several Months (More than 1 month) -- Varios Meses (Mas de 1 mes)	Many months (More than 6 months) -- Muchos meses (Mas de 6 meses)	More than 1 year -- Mas de 1año
Seizures -- Convulsiones	☐	☐	☐	☐	☐	☐	☐
Ringing in your ears -- Zumbido en los oídos	☐	☐	☐	☐	☐	☐	☐
Feeling out of breath or difficulty breathing -- Sensación de falta de aire o dificultad para respirar	☐	☐	☐	☐	☐	☐	☐
Wheezing (high pitched whistling sound made while breathing)a -- Sibilancias	☐	☐	☐	☐	☐	☐	☐
Joint pain -- Dolor en las articulaciones	☐	☐	☐	☐	☐	☐	☐
Muscle aches -- Dolor muscular	☐	☐	☐	☐	☐	☐	☐
Fatigue/tiredness -- Fatiga/cansancio	☐	☐	☐	☐	☐	☐	☐
Problems concentrating -- Problemas de concentración	☐	☐	☐	☐	☐	☐	☐
Vision problems -- Problemas de visión	☐	☐	☐	☐	☐	☐	☐
Poor coordination (ex: Change in balance) -- Poca coordinación (ejemplo: cambios en el balance)	☐	☐	☐	☐	☐	☐	☐

Changes in taste or smell -- Cambios en sabor u olor	☐	☐	☐	☐	☐	☐	☐
Difficulty sleeping or change in sleep patterns since COVID-19 diagnosis -- Dificultad para dormir o cambio en el patrón de sueño desde su diagnóstico de COVID-19	☐	☐	☐	☐	☐	☐	☐
Bedwetting -- Incontinencia nocturna	☐	☐	☐	☐	☐	☐	☐
Fainting -- Desmayos	☐	☐	☐	☐	☐	☐	☐
Other -- Otro	☐	☐	☐	☐	☐	☐	☐

If other, please specify.

Si su respuesta es otro, por favor especificar. ______________________________

For the following symptoms that you experienced after being discharged from hospitalization on [hosp_date_control], was the symptom a new symptom? Or was it a worsening of a preexisting symptom?

De los siguientes síntomas que tu has experimentado después de que le dieran salida de su hospitalización en [hosp_date_control], es este síntoma un nuevo síntoma? O es un síntoma que ya estaba presente antes de este hospitalización y que luego empeoró?

	New symptom since being discharged from hospitalization on [date_admit] (Nuevo síntoma desde que le dieron salida de su hospitalización en [date_admit])	Worsening of a preexisting symptom that started before hospitalization on [date_admit] (Síntoma que empezó antes de su hospitalización en [date_admit] y que luego empeoró)
Seizures -- Convulsiones	○	○
Ringing in your ears -- Zumbido en los oídos	○	○
Feeling out of breath or difficulty breathing -- Sensación de falta de aire o dificultad para respirar	○	○
Wheezing (high pitched whistling sound made while breathing) -- Sibilancias	○	○
Joint pain -- Dolor en las articulaciones	○	○
Muscle aches -- Dolor muscular	○	○

Fatigue/tiredness -- Fatiga/cansancio	○	○
Problems concentrating -- Problemas de concentración	○	○
Vision problems -- Problemas de visión	○	○
Poor coordination (ex: Change in balance) -- Poca coordinación (ejemplo: cambios en el balance)	○	○
Changes in taste or smell -- Cambios en sabor u olor	○	○
Difficulty sleeping or change in sleep patterns since COVID-19 diagnosis -- Dificultad para dormir o cambio en el patrón de sueño desde su diagnóstico de COVID-19	○	○
Bedwetting -- Incontinencia nocturna	○	○
Fainting -- Desmayos	○	○
Other -- Otro	○	○

SECTION 3: Social and Familial History
SECCIÓN 3: Historia Social y Familiar

The current coronavirus (COVID-19) is causing extra stress for many people, including families with children of all ages. We would like to know how things are going for you and your family related to this situation. Please answer the following questions about your experiences and feelings over the last few weeks.
El presente coronavirus (COVID-19) le está causando a muchas personas estrés adicional, incluyendo familias con niños de todas las edades. Nos gustaría poder conocer cómo las cosas han estado para usted y su familia en relación a esta situación. Por favor contestar las siguientes preguntas a cerca de su experiencia y sentimientos en las últimas semanas

Because of COVID-19 related events and changes, I have felt increased stress about:
En relación a los eventos y cambios del COVID-19, me he sentido estresado a cerca de:

	Strongly Disagree -- Muy en desacuerdo	Somewhat Disagree -- Algo en desacuerdo	Neither Agree nor Disagree -- Ni de acuerdo ni en desacuerdo	Somewhat Agree -- Algo en acuerdo	Strongly Agree -- Muy en acuerdo

Food running out or being unavailable -- La comida se acaba o no está disponible	◯	◯	◯	◯	◯
Losing a job or decrease in family income -- Perder el trabajo o disminución del ingreso familiar	◯	◯	◯	◯	◯
Housing or utilities -- Vivienda o servicios públicos	◯	◯	◯	◯	◯
Loss of or limited childcare -- Pérdida o el cuidado limitado de los niños	◯	◯	◯	◯	◯
Taking care of children, including those who are normally in school -- Cuidar a los niños incluyendo a los que van a la escuela	◯	◯	◯	◯	◯
Tension or conflict between household members-- Tensión o conflicto entre los miembros de la familia	◯	◯	◯	◯	◯
Physical health concerns for me or a family member -- Preocupación acerca de mi salud física o de un miembro de la familia	◯	◯	◯	◯	◯
Increased anxiety or depression -- Aumento en la ansiedad o depresión	◯	◯	◯	◯	◯
Reminders of past stressful/traumatic events -- Recuerdos de situaciones estresantes en el pasado/eventos traumáticos	◯	◯	◯	◯	◯
Loss of social connections, social isolation -- Pérdida de conexión social, aislamiento social	◯	◯	◯	◯	◯
Increased tension around school work at home -- Aumento de tensión sobre el trabajo escolar en la casa	◯	◯	◯	◯	◯

How often have you been bothered by each of the following symptoms during the past two weeks?

¿Qué tan seguido, durante las dos ultimas semanas, le han molestado cada uno de los siguientes síntomas?

	0 -- Not at all (En lo absoluto)	1 -- Several days (Varios días)	2 -- More than half the days (Más de la mitad de los días)	3 -- Nearly every day (Aproximadamente cada día)
Feeling down, depressed, irritable, or hopeless? -- Se siente decaído, deprimido, irritable, sin esperanza?	○	○	○	○
Little interest or pleasure in doing things? -- Poco interés o placer en hacer cosas?	○	○	○	○
Trouble falling asleep, staying asleep, or sleeping too much? -- Tiene problema para dormir, permanecer dormido o duerme mucho?	○	○	○	○
Poor appetite, weight loss, or overeating? -- Tiene poco apetito, pérdida de peso o come en exceso?	○	○	○	○
Feeling tired, or having little energy? -- Se siente cansado o tiene poca energía?	○	○	○	○
Feeling bad about yourself-- or feeling that you are a failure, or that you have let yourself or your family down? -- Se siente mal acerca de usted mismo - o se siente que es un fracasado, o que ha decepcionado a usted mismo o a su familia?	○	○	○	○
Trouble concentrating on things like school work, reading, or watching TV? -- Tiene problemas para concentrarse en realizar su trabajo de la escuela, leer o ver televisión?	○	○	○	○

Moving or speaking so slowly that other people could have noticed? Or the opposite-- being so fidgety or restless that you were moving around a lot more than usual? -- Se mueve o siente que habla tan despacio que otras personas lo han notado? O lo contrario - se siente inquieto que se mueve demasiado más de lo usual?	◯	◯	◯	◯

Score: ______________________________

In the past year have you felt depressed or sad most days, even if you felt okay sometimes?

En el pasado se ha sentido deprimido o triste la mayoría de los días, a pesar de que se sienta bien algunas veces?

◯ Yes
◯ No

If you are experiencing any of the problems on this form, how difficult have these problems made it for you to do your work, take care of things at home or get along with other people?

Si usted está experimentado algunos de los problemas de este formulario, ¿qué tan difícil han hecho estos problemas desempeñar su trabajo, dedicarse a los asuntos en su casa o llevarse bien con otras personas?

◯ Not difficult at all -- Sin dificultad
◯ Somewhat difficult -- Alguna dificultad
◯ Very difficult -- Muy difícil
◯ Extremely difficult -- Extremadamente difícil

How often have you been bothered by each of the following symptoms during the past two weeks?

¿Qué tan seguido, durante las dos ultimas semanas, le han molestado cada uno de los siguientes síntomas?

	0 -- Not at all (En lo absoluto)	1 -- Several days (Varios días)	2 -- More than half the days (Más de la mitad de los días)	3 -- Nearly every day (Aproximadamente cada día)
Little interest or pleasure in doing things? -- Siente poco interés o placer en hacer cosas?	◯	◯	◯	◯
Feeling down, depressed, irritable, or hopeless? -- Se siente decaído, deprimido, irritable, o sin esperanzas?	◯	◯	◯	◯

Trouble falling asleep, staying asleep, or sleeping too much? -- Tiene problema para dormir, permanecer dormido o duerme mucho?	◯	◯	◯	◯
Feeling tired, or having little energy? -- Se siente cansado o tiene poca energía?	◯	◯	◯	◯
Poor appetite or overeating? -- Tiene poco apetito o come en exceso?	◯	◯	◯	◯
Feeling bad about yourself-- or feeling that you are a failure, or that you have let yourself or your family down? -- Se siente mal acerca de usted mismo - o se siente que es un fracasado, o que ha decepcionado a usted mismo o a su familia?	◯	◯	◯	◯
Trouble concentrating on things, such as reading the newspaper or watching television? -- Tiene problemas para concentrarse en realizar cosas tales como leer el periódico o ver la televisión?	◯	◯	◯	◯
Moving or speaking so slowly that other people could have noticed? Or the opposite-- being so fidgety or restless that you were moving around a lot more than usual? -- Se mueve o siente que habla tan despacio que otras personas lo han notado? O lo contrario - se siente inquieto que se mueve demasiado más de lo usual?	◯	◯	◯	◯

Score: ______________________________

If you are experiencing any of the problems on this form, how difficult have these problems made it for you to do your work, take care of things at home or get along with other people?

Si usted está experimentado algunos de los problemas de este formulario, ¿qué tan difícil han hecho estos problemas desempeñar su trabajo, dedicarse a los asuntos en su casa o llevarse bien con otras personas?

◯ Not difficult at all -- Sin dificultad
◯ Somewhat difficult -- Alguna dificultad
◯ Very difficult -- Muy difícil
◯ Extremely difficult -- Extremadamente difícil

Over the last two weeks, how often have you been bothered by any of the following problems?

En las últimas dos semanas, ¿qué tan seguido le ha molestado alguno de los siguientes problemas?

	0-- Not at all sure (No estoy seguro)	1-- Several days (Varios días)	2-- Over half the days (Más de la mitad de los días)	3-- Nearly every day (Aproximadamente cada día)
Feeling nervous, anxious, or on edge -- Se siente nervioso, ansioso, o al borde?	○	○	○	○
Not being able to stop or control worrying -- Se siente que no es capaz de detenerse o controlar su preocupación?	○	○	○	○
Worrying too much about different things? -- Se preocupa mucho por diferentes cosas?	○	○	○	○
Trouble relaxing -- Tiene problemas para relajarse	○	○	○	○
Being so restless that it's hard to sit still -- Se siente demasiado inquieto que le es difícil permanecer sentado tranquilo	○	○	○	○
Becoming easily annoyed or irritable -- Le es fácil enojarse o irritarse	○	○	○	○
Feeling afraid as if something awful might happen -- Siente miedo como si algo malo fuera a suceder	○	○	○	○

Score: ______________________________

If you checked off any problems, how difficult have these made it for you to do your work, take care of things at home, or get along with other people?

Si usted está experimentado algunos de los problemas, ¿qué tan difícil han hecho estos problemas desempeñar su trabajo, dedicarse a los asuntos en su casa o llevarse bien con otras personas?

○ Not difficult at all -- Sin dificultad
○ Somewhat difficult -- Alguna dificultad
○ Very difficult -- Muy difícil
○ Extremely difficult -- Extremadamente difícil

Please read each item carefully and mark the response that best describes you.

Por favor lea detenidamente cada uno de los siguientes elementos y seleccione la respuesta que mejor lo describa a usted

	0-- Never (Nunca)	1-- Sometimes (Algunas veces)	2-- Often (A menudo)
Fidgety, unable to sit still -- Nervioso, incapaz de permanecer sentado quieto	○	○	○
Feel sad, unhappy -- Triste, infeliz	○	○	○
Daydream too much -- Sueña despierto demasiado	○	○	○
Refuse to share -- Se rehusa a compartir	○	○	○
Do not understand other people's feelings -- No entiende los sentimientos de los demás	○	○	○
Feel hopeless -- Se siente sin esperanzas	○	○	○
Have trouble concentrating -- Problemas para concentrarse	○	○	○
Fight with other children -- Pelea con otros niños	○	○	○
Down on yourself -- Duro consigo mismo	○	○	○
Blame others for his or her troubles -- Culpa a otros por sus problemas	○	○	○
Seem to be having less fun -- Parece tener menos diversión	○	○	○
Do not listen to rules -- No escucha las reglas	○	○	○
Act as if driven by a motor -- Actúa como impulsado por un motor	○	○	○
Tease others -- Molesta a otros	○	○	○
Worry a lot -- Se preocupa demasiado	○	○	○
Take things that do not belong to him or her -- Toma cosas que no le pertenecen	○	○	○
Distracted easily -- Se distrae fácilmente	○	○	○

Score: ______________________________

In the last year have you seen your primary care provider, either via telehealth or in person?<br><br>En el último año usted ha visto a su proveedor de atención primaria, ya sea por vía telemedicina o en persona?	◯ Yes<br>◯ No
If no, why not? (may check more than one)<br><br>Si su respuesta es no, ¿por qué no? (puede seleccionar más de una)	☐ Wasn't told it was needed -- No me dijeron que era necesario<br>☐ Worried about getting COVID -- Me preocupaba obtener COVID<br>☐ Could not get an appointment -- No pude conseguir cita<br>☐ Too busy -- Demasiado ocupado<br>☐ Didn't feel it was necessary. -- Sentí que no era necesario<br>☐ Other -- Otro
If other, please specify.<br><br>Si su respuesta es otro, por favor especificar	__________________________________
If yes, how many times?<br><br>Si su respuesta es sí, ¿cuántas veces?	____________________________
Have you received the SARS-CoV-2 vaccine?<br><br>¿Ha recibido la vacuna para SARS-COV-2?	◯ Yes<br>◯ No
If no, please check off the reason why. (may check more than one)<br><br>Si su respuesta es no, por favor seleccionar la razón (puede seleccionar más de una)	☐ Don't feel it is needed -- Sentí que no era necesario<br>☐ Concerned about side effects -- Me preocupaba acerca de los efectos secundarios<br>☐ Too much work required -- Requería demasiado trabajo<br>☐ Tried to, but was unable to get -- Lo intenté, pero no lo pude obtener<br>☐ Other -- Otro
If other, please specify.<br><br>Si su respuesta es otro, por favor especificar	__________________________________
