# Supplementary material for: Persistent physical and mental health effects of COVID-19 hospitalization
Source: Front Pediatr. 2026 Jul 30;14:1710900. doi: 10.3389/fped.2026.1710900 (PMC13469453; doi:10.3389/fped.2026.1710900)
Supplement: Supplementary file 5 [file Table1.docx]

|  | **PHQ-9 (n=31)** | | **GAD-7 (n=31)** | | **PSC-17 (n=64)** | |
| --- | --- | --- | --- | --- | --- | --- |
| Binary Predictors | | | | | | |
|  | Median (IQR) | p | Median (IQR) | p | Median (IQR) | p |
| **Sex** | | | | | | |
| Male | 1 (0–8) | 0.66 | 0 (0–6) | 0.41 | 2 (0–10) | 0.56 |
| Female | 5 (0–7) |  | 5 (0–9) |  | 1 (0–4) |  |
| **ICU Admission** | | | | | | |
| Yes | 2 (0–5) | 0.32 | 2 (0–4) | 0.43 | 3 (1–6) | 0.25 |
| No | 5 (0–9) |  | 3 (0–10) |  | 1 (0–6) |  |
| **Variant Wave** | | | | | | |
| Early (Original/Alpha) | 5 (1–10) | 0.11 | 4 (0–9) | 0.21 | 1 (0–4) | 0.20 |
| Late (Omicron) | 0 (0–3) |  | 0 (0–3) |  | 6 (1–6) |  |
| **MIS-C** | | | | | | |
| Yes | 1 (0–2) | 0.34 | 2 (0–4) | 0.44 | 1 (0–3) | 0.72 |
| No | 5 (0–9) |  | 3 (0–10) |  | 2 (0–6) |  |
|  | | | | | | |
| Continuous Predictors | | | | | | |
|  | **PHQ-9 (n=31)** | | **GAD-7 (n=31)** | | **PSC-17 (n=64)** | |
|  | rho | p | rho | p | rho | p |
| **Age** | 0.23 | 0.22 | 0.19 | 0.30 | 0.03 | 0.85 |
| **Length of Stay** | −0.01 | 0.97 | −0.06 | 0.75 | 0.04 | 0.75 |

**Supplemental Table 1. Bivariate Analysis of Potential Confounders.** Bivariate analysis revealed no statistically significant associations between any of the potential confounders examined and scores on the PHQ-9, GAD-7, or PSC-17 (all p ≥ 0.05). IQR = interquartile range; PHQ-9 = Patient Health Questionnaire-9; GAD-7 = Generalized Anxiety Disorder-7; PSC-17 = Pediatric Symptom Checklist-17; MIS-C = Multisystem Inflammatory Syndrome in Children. PHQ-9 and GAD-7 were completed by cases only. PSC-17 was completed by cases and controls (n=64). p-values for categorical variables derived from Mann-Whitney U test; rho from Spearman's rank correlation.
